# *Dechloromonas parva* sp. nov., a Member of the Family *Azonexaceae*

**DOI:** 10.4014/jmb.2602.02044

**Published:** 2026-08-24

**Authors:** Min-gyung Baek, Ji-Hyeon Son, Hana Yi, Sangcheol Park

**Affiliations:** 1 Institute for Biomaterials, Korea University, Seoul 02841, Republic of Korea; 2 Integrated Biomedical and Life Science, Graduate School, Korea University, Seoul 02841, Republic of Korea; 3 Interdisciplinary Program in Precision Public Health, Korea University, Seoul 02841, Republic of Korea; 4 School of Biosystems and Biomedical Sciences, Korea University, Seoul 02841, Republic of Korea; 5 Graduate School of Medical Science and Engineering, KAIST, Daejeon 34141, Republic of Korea

**Keywords:** *Dechloromonas*, *Ferribacterium*, *Quatrionicoccus*, *Azonexaceae*

## Abstract

A *Dechloromonas*-like bacterial strain, designated HYN0024^T^, was isolated from lake water, and its taxonomic status was characterized in this study. Although the bacterium was isolated from an aerobic water sample, its cells were facultative anaerobes utilizing Fe(III) and oxygen as terminal electron acceptors. Phylogenetic analysis based on both genome and 16S rRNA gene sequences indicated that strain HYN0024^T^ belongs to the family *Azonexaceae* and forms a monophyletic clade with the type strains of *Ferribacterium limneticum*, *Quatrionicoccus australiensis*, and ‘*Dechloromonas aromatica*’. However, the type strains of the genera *Ferribacterium* and *Quatrionicoccus* are currently unavailable from any public culture collections. Among the available type strains with validly published names, *Dechloromonas hortensis* shared the highest 16S rRNA gene sequence similarity (98.35%) with strain HYN0024^T^, but their average nucleotide identity (80.13%) was below the threshold for species delineation. The biochemical and physiological features also supported the distinctiveness of the isolate from previously known species. The 16S rRNA gene sequence similarity, genomic relatedness, phylogenetic tree topology, and phenotypic characteristics supported the taxonomic independence of the isolate as a novel species in the genus *Dechloromonas*. Based on the phylogenetic and polyphasic taxonomic data presented in this study, we propose a novel species, *Dechloromonas parva* sp. nov., for strain HYN0024^T^ (= KACC 19179^T^ = NBRC 112737^T^).

## Introduction

The genus *Dechloromonas*, belonging to the family *Azonexaceae*, was proposed with the type species *D. agitata* in 2001 [1]. The genus is known as Gram-stain-negative, facultative anaerobic rods with a flagellum [1]. Members of the genus *Dechloromonas* have been isolated from a pulp sludge at a paper mill plant [1], the gut of an earthworm [2], polluted and pristine sites [3], and wetland soil [4]. Currently, three validly published names (*D. agitata*, *D. denitrificans*, and *D. hortensis*) and two not validly published names (‘*D. aromatica*’, whose type strain is not accessible in public culture collections, and ‘*D. hankyongensis*’) are listed as child taxa in this genus according to the List of Prokaryotic names with Standing in Nomenclature (LPSN) (http://lpsn.dsmz.de/). In addition, this genus includes three Candidatus species (*Candidatus* Dechloromonas occultata, *Ca*. D. phosphoritropha, and *Ca*. D. phosphorivorans) which are known to inhabit lake sediment [5] and wastewater treatment systems [6].

The family *Azonexaceae* encompasses the genera *Azonexus*, *Dechloromonas*, *Ferribacterium*, and *Quatrionicoccus*. In 16S rRNA gene analyses, the genera *Quatrionicoccus* and *Ferribacterium* form branches within the radiation of the genus *Dechloromonas* [2, 4, 5, 7-15]. Thus, the taxonomic relationship of these three genera is worth studying. However, *Quatrionicoccus australiensis* NCIMB 13738^T^, the only type strain of this genus, has been lost in public culture collections and is no longer accessible. Similarly, *Ferribacterium limneticum* CdA-1^T^, the sole type strain of this genus, is also reported as no longer available [2]. We also tried to cultivate *Ferribacterium limneticum* ATCC 700589^T^, but failed to revive the stock under the recommended anaerobic culture conditions. Thus, it is fair to say that the family *Azonexaceae* currently encompasses only two available genera, namely *Azonexus* and *Dechloromonas*.

Here, we report a new *Dechloromonas*-like strain, designated HYN0024^T^, which was isolated from a freshwater environment. In order to determine the taxonomic position of the new isolate, its polyphasic taxonomic characteristics including complete genome sequencing, chemotaxonomic profiles, and biochemical/physiological features were thoroughly examined.

## Materials and Methods

### Isolation

Strain HYN0024^T^ was isolated from freshwater in Hapcheon lake (N35°31´40˝ E128°01´20˝), Gyeongsang-do, South Korea. For serial dilution and spread plating, sterilized distilled water and R2A agar (BD, USA) were used. The isolate was cultured aerobically at 25°C on R2A agar, and stored at -80°C in a glycerol suspension (20% in distilled water, w/v). For the comparative taxonomic study, *Dechloromonas hortensis* DSM 15637^T^ was selected as the closest relative type strain and used as an experimental control.

### 16S rRNA Gene Phylogeny

The 16S rRNA gene was amplified using primers 27F and 1492R and sequenced using 27F, 518F, 907R, and 1492R as described previously [16]. The determined sequence was identified using the EzBioCloud server [17], aligned with the EzEditor2 program [18] and subjected to phylogenetic analyses using the MEGA 7.0 program [19]. A Maximum-likelihood tree using the general time reversible model, a Neighbor-joining tree using the Kimura 2-parameter model, and a Maximum-parsimony tree using the subtree-pruning-regrafting model were constructed. Bootstrap analysis (1,000 replications) was used to evaluate tree topologies.

### Genome Analyses

Whole-genome sequencing was performed using the PacBio RS II system (Pacific Biosciences) and the resultant sequence was assembled using PacBio SMRT Analysis v2.3.0. The annotation was processed by the NCBI Prokaryotic Genome Annotation Pipeline [20], and the complete genome sequence of strain HYN0024^T^ was registered in GenBank under the accession number CP031842. A genome-based phylogenetic tree was inferred using the UBCG pipeline version 3.0 [21]. The genomic relatedness between the isolate and the closely related type strain was estimated by calculating the average nucleotide identity (ANI) in OrthoANI [22], the average amino acid identity (AAI) [23], and the genome-to-genome distance using the GGDC calculator [24]. Metabolic properties were predicted using the KEGG pathway analysis tool implemented in the RAST server [25].

### Phenotype Characterization

The morphological, physiological, and biochemical characteristics were examined under the same laboratory conditions as described previously [16]. Colonial morphology was examined using R2A agar at 25°C for 3−7 days. Cellular morphology was observed by transmission electron microscopy after negative staining with 2% phosphotungstic acid hydrate.

Growth on different media was tested under aerobic conditions on R2A agar, Tryptic Soy agar (TSA; BD), MacConkey agar (BD), and Nutrient agar (NA; BD). Anaerobic growth was evaluated by incubating the cultures in an AnaeroPack-Anaero system (Mitsubishi Gas Chemical Co., Japan) for 7 days at 25°C on the following media: R2A with and without potassium nitrate (0.084%), potassium chlorate (10 mM), sodium fumarate (10 mM), or Fe(III)-pyrophosphate (0.3%).

The utilization of electron donors and acceptors was assessed using a basal medium composed of yeast extract (0.05%), proteose peptone no. 3 (0.05%), casamino acids (0.05%), dipotassium phosphate (0.03%), magnesium sulfate (0.005%), and agar (1.5%). To evaluate electron donor preference, acetate, propionate, pyruvate, and starch (each at 10 mM) were tested with Fe(III)-pyrophosphate (10 mM) or oxygen as the terminal electron acceptor. Conversely, the utilization of electron acceptors was examined using pyruvate (10 mM) as the electron donor, with nitrate, chlorate, Fe(III)-pyrophosphate, and fumarate (each at 10 mM) as candidates. Anaerobic conditions for the tests were maintained using the GENbox Anaero system (bioMérieux, France) according to the manufacturer’s instructions.

The temperature range (4°C, 10°C, 15°C, 20°C, 25°C, 30°C, 35°C, and 40°C) for growth was determined for a week using R2A agar. The pH range (pH 6.0−9.0 with 0.5 pH unit increments) was tested using R2A agar with one of the following buffers: 1 M MES (pH 6.0−6.5), 0.1 M HEPES (pH 7.0−7.5), 1 M Tris (pH 8.0−8.5), or 1 M Na_2_CO_3_ (pH 9.0). To investigate tolerance to NaCl, R2A agar adjusted to 0−5% (w/v) with 1% increments of NaCl concentration was used. Enzyme activities were examined using the API ZYM and API 20 NE kits (bioMérieux) at 25°C for 3 days under aerobic conditions.

To analyze fatty acids, strains HYN0024^T^ and *D. hortensis* DSM 15637^T^ were grown on R2A aerobically at 25°C for 7 and 3 days, respectively. Extraction and separation of fatty acid methyl esters by gas chromatography (Agilent Technologies) were performed according to the manufacturer’s instructions [26]. The Sherlock method of the Microbial Identification System (MIDI) version 6.3 and the TSBA 6.21 databases were used for fatty acid identification. Polar lipids were extracted and separated by two-dimensional chromatography on silica gel TLC plates, and identified by spraying with detection reagents: molybdophosphoric acid (for total lipids; Sigma), Dragendorff’s reagent (for phosphatidylcholine; Sigma), 1-naphthol-sulfuric acid (for glycolipids; Sigma), ninhydrin (for aminolipids; Sigma), and molybdenum blue reagent (for phospholipids; Sigma) [27].

## Results

### Phylogenetic Analysis of the 16S rRNA Gene Sequences

**Fig. 1:**
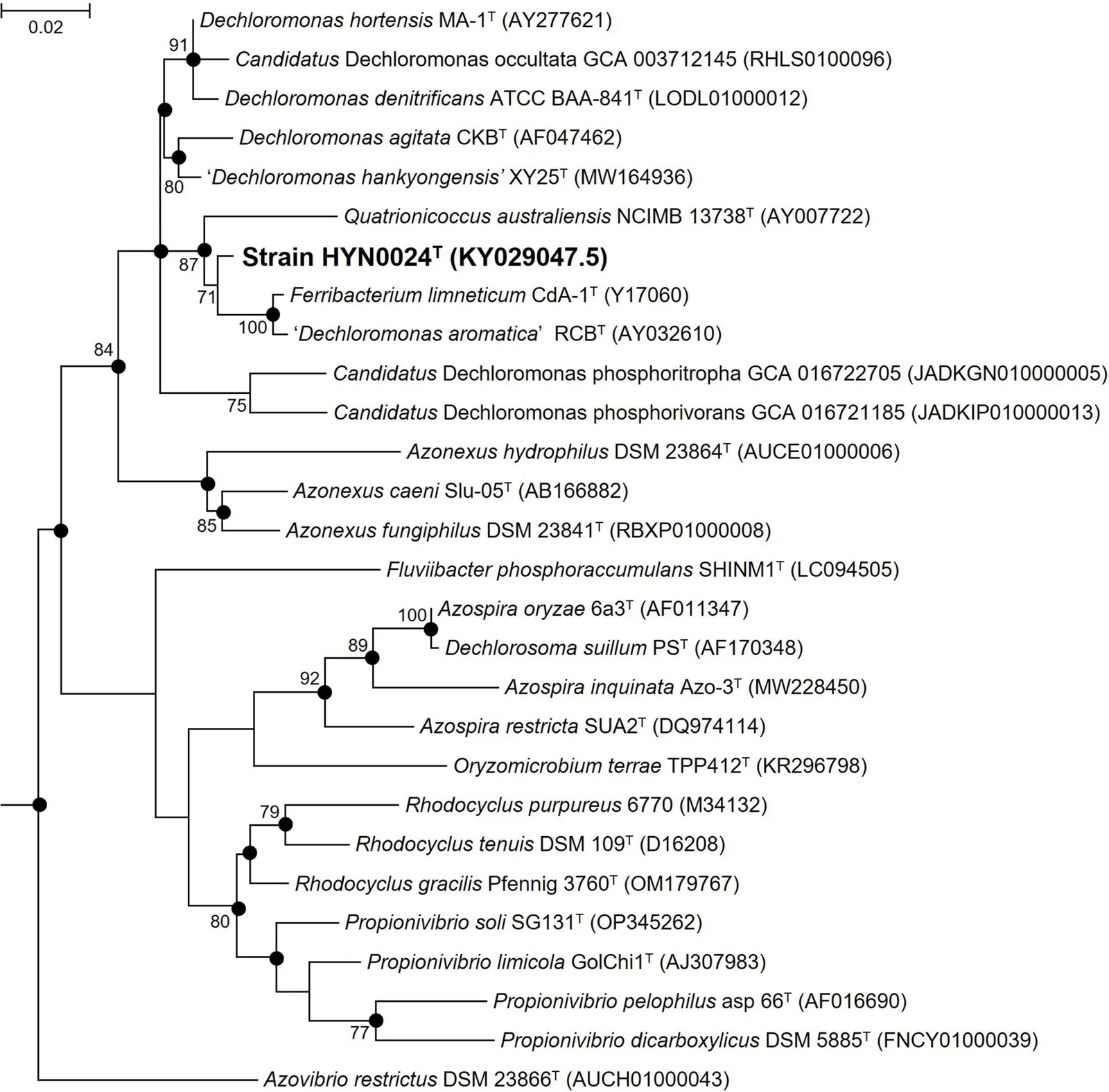
Maximum-likelihood tree based on 16S rRNA gene sequence showing the phylogenetic position of strain HYN0024^T^ in the family *Azonexaceae*. The numbers at the nodes are percentages of bootstrap support (>70%) based on 1,000 resampled data sets. Filled circles indicate nodes also obtained in both Neighbor-joining and Maximum-parsimony trees. *Nitrosospira lacus* APG3^T^ (KC477402) was used as an outgroup. Scale bar, 0.02 substitutions per nucleotide position.

Strain HYN0024^T^ exhibited the highest 16S rRNA gene sequence similarity to *D. hortensis* MA-1^T^ (98.35%) and ‘*D. aromatica* RCB^T^’ (98.35%), followed by *F. limneticum* CdA-1^T^ (98.21%), *D. denitrificans* ED1^T^ (97.87%), ‘*D. hankyongensis*’ XY25^T^ (97.46%), *Q. australiensis* NCIMB 13738^T^ (97.07%), and *D. agitata* CKB^T^ (96.91%). All 16S rRNA gene trees inferred in this study placed strain HYN0024^T^ within the family *Azonexaceae*, forming a monophyletic clade with the type strains of *Ferribacterium limneticum*, *Quatrionicoccus australiensis*, and ‘*Dechloromonas aromatica*’ (Fig. 1). Based on the sequence similarity and tree topology, the type strains of these three taxa would ideally serve as experimental controls. However, as noted in the introduction, they are currently unavailable from any public culture collections. Furthermore, genomic resources for these taxa are extremely limited: while a single genome for *F. limneticum* is available, it does not represent the type strain, and no genome sequence exists for *Q. australiensis*. These limitations precluded comprehensive genomic and phenotypic comparisons with these three closely related type strains. Consequently, among the available type strains with validly published names, *Dechloromonas hortensis* MA-1^T^, which shares the highest sequence similarity (98.35%) with the isolate, was selected as the experimental control for this study.

### Genomic Characteristics

The genome of strain HYN0024^T^ was assembled into a circular chromosome with a total length of 3.26 Mb. The genomic G+C content was 59.4%. Of the 3,144 genes, 3,032 are protein-coding genes and 71 are RNA genes (9 rRNAs, 58 tRNAs, and 4 ncRNAs; Table S1). The genome encodes three copies of the rRNA operon, but sequence variation within the three 16S rRNA gene copies was not observed.

The genome tree, inferred using 92 bacterial core genes shared by the isolate and its closest relatives, supported the status of strain HYN0024^T^ as a novel species within the genus *Dechloromonas* (Fig. S1). The ANI value between strain HYN0024^T^ and its closest type strain, *D. hortensis* MA-1^T^, was 80.13%, which is well below the threshold (95−96%) for bacterial species delineation. Digital DNA-DNA hybridization (dDDH) between strain HYN0024^T^ and *D. hortensis* MA-1^T^ was 23.1%, a value significantly below the 70% cut-off for species circumscription. The corresponding AAI value was 80.3%, which is also below the generally accepted species threshold (95−96%), further confirming their genomic distinctiveness. Additionally, comparisons with the genome of a non-type strain, *F. limneticum* 77, also showed low genomic relatedness (AAI 87.1%, ANI 83.98%, and dDDH 27.1%).

Based on the KEGG pathway annotations, the TCA cycle and the Embden-Meyerhof pathway were present. Genes required for assimilatory nitrate reduction, nitrogen fixation, and denitrification were observed. Biosynthesis pathways for threonine, cysteine, valine, isoleucine, leucine, lysine, arginine, proline, tryptophan, phenylalanine, tyrosine, and glutathione were present. Although gene sets for flagellar structural proteins were predicted in the genome of strain HYN0024^T^, several genes—including *fliK*, *flhE*, *flgO*, *flgP*, *flgQ*, *flgT*, *motX*, *motY*, *fliT*, *motC*, *motD*, *fliY*, *fliZ*, *flrA*, *flhD*, and *flhC*— were deficient. Despite the absence of these genes, a single polar flagellum was observed via electron microscopy. This apparent discrepancy may be attributed to limitations in genome annotation, significant sequence divergence of flagellar genes from known references, or the presence of alternative gene organizations and functional analogues. Xenobiotics biodegraded by this bacterium include catechol and propanoyl-CoA.

### Phenotypic Characteristics

Cells were motile with a single polar flagellum (Fig. S2). Strain HYN0024^T^ grew aerobically on R2A agar but did not grow in R2A broth. No other conventional bacterial media tested in this study (TSA, MacConkey, or NA) supported the growth of strain HYN0024^T^. The maximum colony size of HYN0024^T^ was less than 1 mm on R2A under aerobic conditions, which was much smaller than the 2.5 mm colonies of *D. hortensis* DSM 15637^T^. Under anaerobic conditions, the colonial size was even smaller. The growth temperature range was 15−30°C (optimum, 25°C) and the pH range was 7.0−8.0 (optimum, pH 7.5). The strain grew well in the absence of NaCl, but not in the presence of 1% (w/v) NaCl or higher. Strain HYN0024^T^ utilized pyruvate, but not acetate or propionate. The isolate utilized oxygen and Fe(III)-pyrophosphate as the terminal electron acceptor, but not nitrate, chlorate, or fumarate.

In the API 20NE and ZYM kits, no positive reaction was observed. As stated above, strain HYN0024^T^ is a fastidious bacterium that exhibits highly restricted growth on standard chemical formulations; it grew exclusively on R2A agar and failed to grow in standard liquid broths or alternative conventional media (such as TSA, NA, and MacConkey). The completely negative profiles in the API 20NE and API ZYM kits are attributed to the inadequacy of the manufacturers' standard inoculation suspending media, which failed to support the metabolic activity or survival of this specific strain during the incubation period. To address this, we attempted extended incubation times (up to 7 days) and alternative suspension buffers tailored to R2A composition, but no significant colorimetric shifts were observed.

The major cellular fatty acids (>10% of the total fatty acids) in strain HYN0024^T^ were Summed Feature 3 (C_16:1_ *ω*7*c*/C_16:1_ *ω*6*c*; 56.7%), C_16:0_ (14.4%), and C_12:0_ (10.8%) (Table 2). Both strain HYN0024^T^ and *D. hortensis* DSM 15637^T^ possessed Summed Feature 3 as the most predominant fatty acid (56.7% and 50.9%, respectively), and C_16:0_ as the second predominant fatty acid (14.4% and 25.2%, respectively). However, C_12:0_, the third predominant fatty acid in strain HYN0024^T^ (10.8%), was detected only as a minor component in *D. hortensis* DSM 15637^T^ (1.2%).

The major polar lipids detected in strain HYN0024^T^ were phosphatidylethanolamine (PE), phosphatidylglycerol (PG), diphosphatidylglycerol (DPG), two glycolipids (GL1-2), and ten unidentified phospholipids (PL1-10). Additionally, an aminoglycolipid (AGL), phosphoglycolipid (PGL), one unidentified aminolipid (AL1), and six unidentified lipids (L1-6) were also detected as minor and occasional polar lipids (Fig. S3).

The morphological, physiological, and biochemical features of strain HYN0024^T^ were compared with those of related taxa based on both experimental results and published descriptions (Table 1). The cellular fatty acid composition is summarized separately in Table 2.

## Conclusion

In the 16S rRNA gene phylogenetic trees, the genera *Ferribacterium* and *Quatrionicoccus* indeed form a monophyletic lineage nested within the radiation of the genus *Dechloromonas*. This topological nesting strongly implies that the current genus-level boundaries within the family *Azonexaceae* may be over-split and warrant taxonomic reclassification or amalgamation into a single emended genus *Dechloromonas*. However, because the type strains for both *Ferribacterium limneticum* and *Quatrionicoccus australiensis* have been lost and are completely inaccessible from public repositories, formal reclassification cannot be performed in the current study.

The distinct phylogenetic position and low (<98.7%) 16S rRNA gene sequence similarity demonstrated the novel genomic species status of strain HYN0024^T^. The phenotypic properties of the new isolate were also distinct from those of its closest neighbor species, characterized by higher amounts of C_12:0_ fatty acids, an inability to grow on NA, and a lack of oxidase activity. Their profiles for electron donor and acceptor utilization were also distinct. Therefore, we conclude that the test strain represents a novel species of the genus *Dechloromonas*, for which we propose the name *Dechloromonas parva* sp. nov. (type strain HYN0024^T^ = KACC 19179^T^ = NBRC 112737^T^).

### Description of *Dechloromonas parva* sp. nov.

*Dechloromonas parva* (par’va. L. fem. adj. *parva*, small).

Cells are Gram-stain-negative and rod-shaped (approximately 0.4−0.8 μm wide and 1.3-3.1 μm long). Cells are motile with a single polar flagellum. Colonies are small (up to 1 mm in diameter), flat, circular with entire margins, and yellow on R2A agar. Grows on R2A, but not on TSA, MacConkey, or NA. Grows at 15−30°C (optimum, 25°C), a pH of 7.0−8.0 (optimum, 7.5). Grows in the absence of NaCl, but not in the presence of 1% (w/v) NaCl or higher. Uses pyruvate as an electron donor, but not acetate or propionate. Uses O_2_ and Fe(III)-pyrophosphate as terminal electron acceptors, but not nitrate, chlorate, or fumarate. Does not possess catalase or oxidase activities. Possesses Summed Feature 3 (C_16:1_ *ω*7*c*/C_16:1_ *ω*6*c*), C_16:0_, and C_12:0_ as major fatty acids. Possesses PE, PG, DPG, GLs, and PLs as major polar lipids.

The type strain HYN0024^T^ (= KACC 19179^T^ = NBRC 112737^T^) was isolated from freshwater collected from Hapcheon Lake, Gyeongsang-do, South Korea. The GenBank accession numbers for the 16S rRNA gene sequence and the complete genome sequence of the type strain are KY029047 and CP031842, respectively. The genome of the type strain is a 3.26 Mb circular chromosome with a DNA G+C content of 59.4%.

## Supplemental Materials

Supplementary data for this paper are available on-line only at http://jmb.or.kr.



## Figures and Tables

**Table 1:** **Comparative phenotypic characteristics of strain HYN0024^T^ and related taxa.** Strains: 1, strain HYN0024^T^; 2, *Dechloromonas hortensis* DSM 15637^T^; 3, *Ferribacterium limneticum* CdA-1^T^; 4, *Dechloromonas agitata* CKB^T^; 5, *Quatrionicoccus australiensis* NCIMB 13738^T^. Abbreviations: +, positive; w, weakly positive; -, negative; ND, not detected

Characteristic	1	2	3^a^	4^b^	5^c^
Morphology (cell diameter)	Rod-shaped (0.4−0.8 μm)	Rod-shaped (0.3−0.9 μm)	Rod-shaped (1.4−2.0 μm)	Short rod (0.5−2.0 μm)	Coccoid cells (2.2−4.5 μm)
Motility	+	+	+	+	-
Optimum growth temperature (°C)	25	30	ND	35	25-30
Optimum growth pH	7.5	7.2	ND	7.5	7.5-8.5
Oxidase	-	w	ND	ND	-
Catalase	-	-	ND	ND	+
Electron acceptor	Fe(III)-pyrophosphate, oxygen	Fe(III)-pyrophosphate, oxygen, nitrate, fumarate, chlorate	Fe(III)-pyrophosphate, nitrate, fumarate	nitrate, perchlorate, oxygen	ND
Electron donor	pyruvate	acetate, propionate, pyruvate	acetate, formate, lactate, benzoate	acetate, propionate, butyrate, lactate, succinate, fumarate, malate, Fe(II)-pyrophosphate	ND

^a^Data from Cummings *et al.* [11]. ^b^Data from Achenbach *et al.* [1]. ^c^Data from Maszenan *et al.* [28].

**Table 2:** **Cellular fatty acid composition (%) of strain HYN0024^T^ and closely related type strains.** Strains: 1, strain HYN0024^T^; 2, *Dechloromonas hortensis* DSM 15637^T^; 3, *Dechloromonas agitata* CKB^T^; 4, *Dechloromonas denitrificans* ATCC BAA-841^T^; 5, *Dechloromonas hankyongensis* XY25^T^. Abbreviations: +, positive; w, weakly positive; -, negative; tr, trace amounting (<0.5%); ND, not detected

Major fatty acids	1	2	3^b^	4^c^	5^d^
C_12:0_	10.8	1.2	7.9	2.5	8.1
C_14:0_	4.2	5.5	2.1	1.1	1.7
C_16:0_	14.4	25.2	25.5	21.3	17.4
C_10:0_ 3-OH	6.9	6.2	4.6	3.4	4.7
C_12:1_ 3-OH	-	4.9	tr	2.7	tr
C_13:0_ 3-OH	6.6	-	ND	ND	ND
Summed Feature 2^a^	3.3	-	2.0	tr	2.6
Summed Feature 3^a^	56.7	50.9	40.5	58.4	60.8
DNA G+C content (%)	59.4	61.4	63.5	61.2	62.9

^a^Summed Feature 2 was listed as C_12:0_ aldehyde/unknown 10.928; Summed Feature 3 was listed as C_16:1_ *ω*7*c*/C_16:1_ *ω*6*c*. ^b^Data from Achenbach *et al.* [1]. ^c^Data from Horn *et al.* [2]. ^d^Data from Kim *et al.* [4].
