# Supplementary material for: Dechloromonas parva sp. nov., a Member of the Family Azonexaceae
Source: J Microbiol Biotechnol. 2026 Aug 24;36:e2602044. doi: 10.4014/jmb.2602.02044 (PMC13550009; doi:10.4014/jmb.2602.02044)

J. Microbiol. Biotechnol. 2026. 36: e2602044

https://doi.org/10.4014/jmb.2602.02044

Supplementary Figure and Tables

Dechloromonas parva sp. nov., a member of the family *Azonexaceae*

Min-gyung Baek[1], Ji-Hyeon Son[2,3], Hana Yi[1,2,3,4]*, and Sangcheol Park[5]*

[1] Institute for Biomaterials, Korea University, Seoul, Korea

[2] Integrated Biomedical and Life Science, Graduate School, Korea University, Seoul, Korea

[3] Interdisciplinary Program in Precision Public Health, Korea University, Seoul, Korea

[4] School of Biosystems and Biomedical Sciences, Korea University, Seoul, Korea

[5]Graduate School of Medical Science and Engineering, KAIST, Daejeon, Korea

*Corresponding author and email address

Prof. Hana Yi

(E-mail): hanayi@korea.ac.kr

Dr. Sangcheol Park

(E-mail): cukucu123@gmail.com

Table S1. Genome sequence features of strain HYN0024^{T}.

Features	Strain HYN0024^{T}
BioProject	PRJNA476041
Accession number	CP031842
Sequencing Technology	RSII PacBio
Assembly method	PacBio SMRT Analysis v. 2.3
Genome coverage	312.55x
N50	3,258,300
Number of contigs	1
Genome size (bp)	3,258,300
DNA GC content (mol%)	59.4
Total genes	3,144
Protein coding genes	3,032
rRNAs	9
tRNAs	58
ncRNAs	4
Pseudo genes	41
rRNA operon copy numbers	3
16S rRNA copy number variation (%)	0

Fig. S1. Maximum-likelihood tree inferred using 92 bacterial core gene sequences showing the phylogenomic position of strain HYN0024^{T} within the family *Azonexaceae*. The numbers at the nodes are bootstrap support percentages (>70%) based on 1000 resampled datasets *Nitrosospira lacus* APG3^{T} (KC477402) was used as an outgroup. Bar, 0.05 nucleotide substitutions per position.

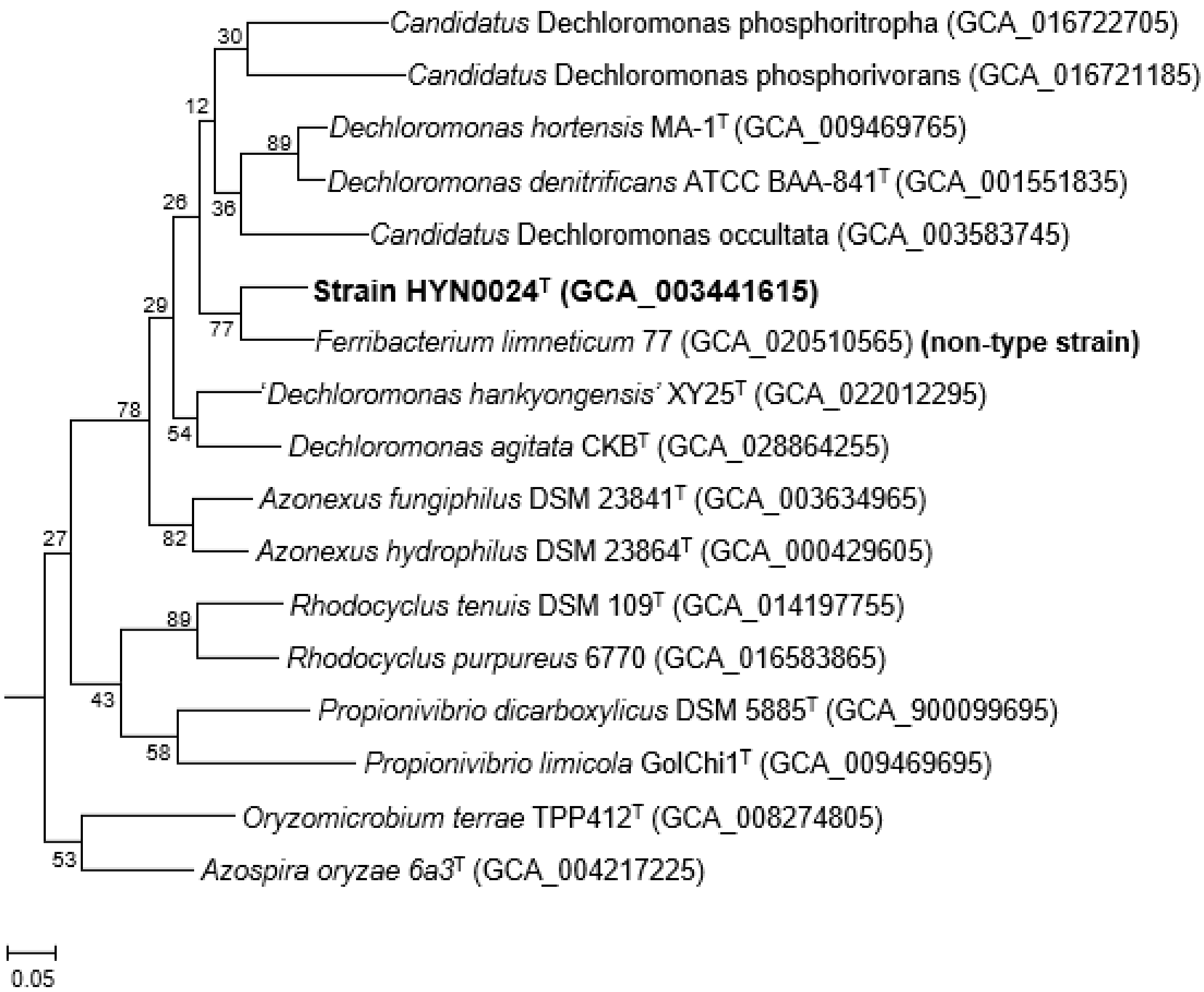

Fig. S2. Transmission electron micrograph of strain HYN0024^T cultured on R2A aerobically at 25°C for 7 days. Bar, 2 µm.

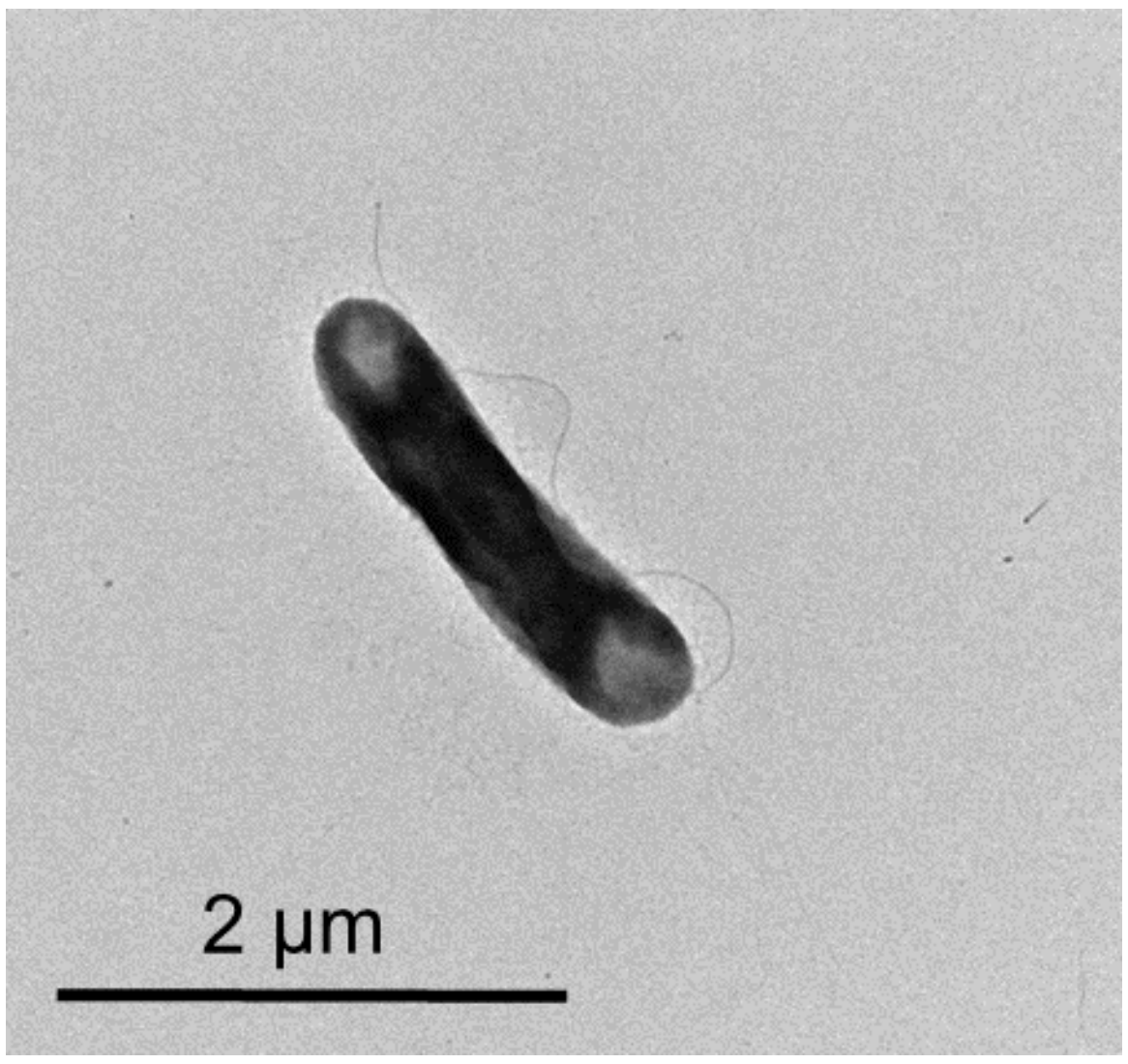

Fig. S3. Two-dimensional thin-layer chromatograms of total polar lipids.

DPG, diphosphatidylglycerol; PG, phophatidylglycerol; PE, phosphatidylethanolamine; GL, glycolipid; PL, unidentified phospholipid; AGL, aminoglycolipid; PGL, phophoglycolipid; AL, unidentified aminolipid; L, unidentified lipid.

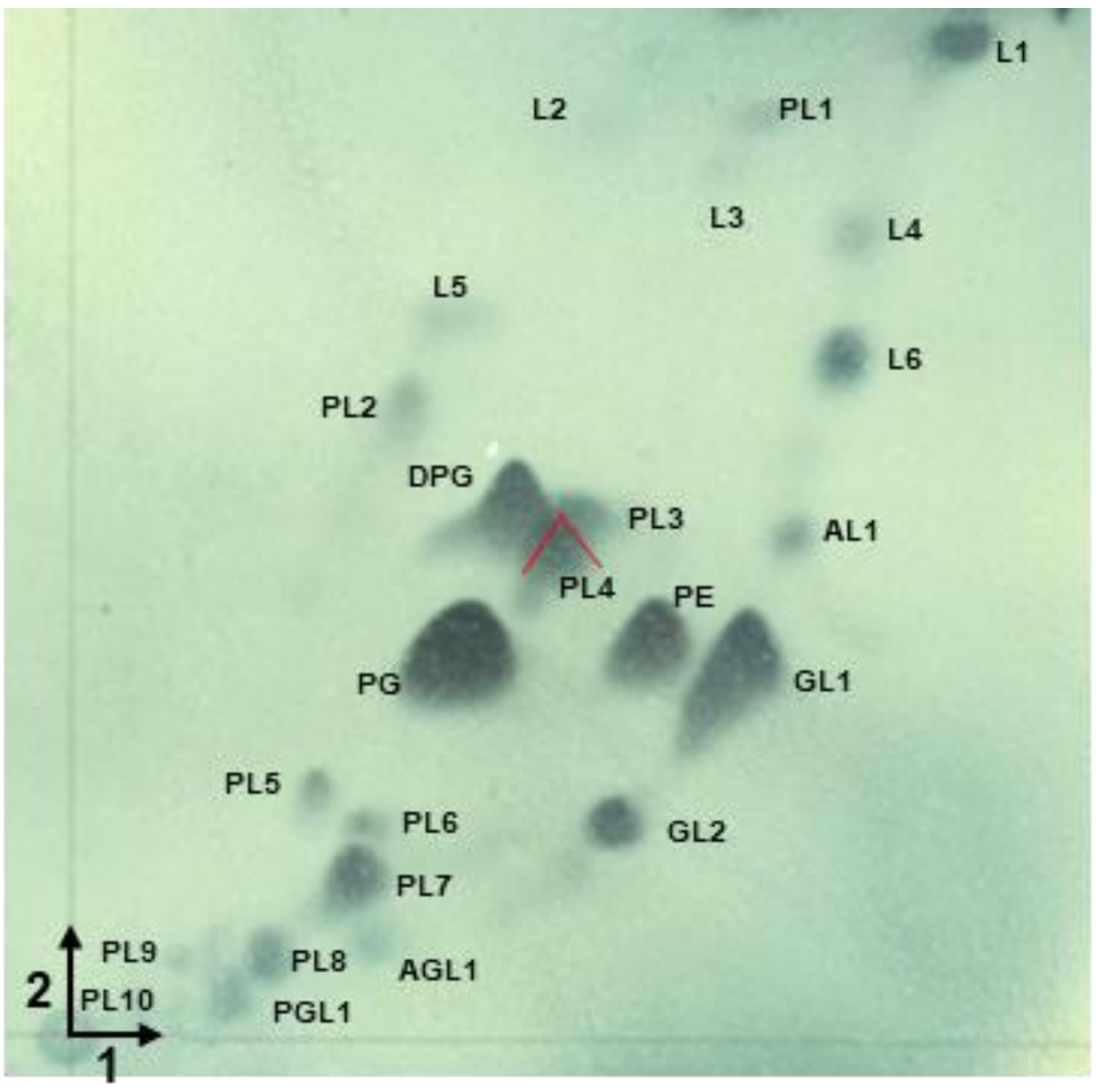

Supplement: Supplementary file 1 [file jmb-36-e2602044-supple.pdf]
